# Apoplastic barriers of *Populus* × *canescens* roots in reaction to different cultivation conditions and abiotic stress treatments

**DOI:** 10.1007/s44154-023-00103-3

**Published:** 2023-07-21

**Authors:** Paul Grünhofer, Ines Heimerich, Lena Herzig, Svenja Pohl, Lukas Schreiber

**Affiliations:** Department of Ecophysiology, Institute of Cellular and Molecular Botany, University of Bonn, Kirschallee 1, 53115 Bonn, Germany

**Keywords:** Hydroponics, Aeroponics, Soil, Abscisic acid (ABA), Oxygen deficiency, Poplar root suberin

## Abstract

*Populus* is an important tree genus frequently cultivated for economical purposes. However, the high sensitivity of poplars towards water deficit, drought, and salt accumulation significantly affects plant productivity and limits biomass yield. Various cultivation and abiotic stress conditions have been described to significantly induce the formation of apoplastic barriers (Casparian bands and suberin lamellae) in roots of different monocotyledonous crop species. Thus, this study aimed to investigate to which degree the roots of the dicotyledonous gray poplar (*Populus* × *canescens*) react to a set of selected cultivation conditions (hydroponics, aeroponics, or soil) and abiotic stress treatments (abscisic acid, oxygen deficiency) because a differing stress response could potentially help in explaining the observed higher stress susceptibility. The apoplastic barriers of poplar roots cultivated in different environments were analyzed by means of histochemistry and gas chromatography and compared to the available literature on monocotyledonous crop species. Overall, dicotyledonous poplar roots showed only a remarkably low induction or enhancement of apoplastic barriers in response to the different cultivation conditions and abiotic stress treatments. The genetic optimization (e.g., overexpression of biosynthesis key genes) of the apoplastic barrier development in poplar roots might result in more stress-tolerant cultivars in the future.

**Supplementary Information:**

The online version contains supplementary material available at 10.1007/s44154-023-00103-3.

## Introduction

Although poplar trees are abundantly cultivated in agroforestry due to their rapid growth (Dillen et al. 2010) and thus are economically very important (Sannigrahi and Ragauskas 2010; Taylor 2002), many species of the genus *Populus* are known for their high sensitivity towards water deficit, drought, and salt accumulation (Bolu and Polle 2004; Larchevêque et al. 2011; Robison and Raffa 1998; Silim et al. 2009). In turn, these undesired traits significantly affect plant productivity and limit the biomass yield, for example in short rotation coppice cultures (Al Afas et al. 2006; Allen et al. 1999). A noteworthy exception exhibiting remarkable stress tolerances towards high light, extreme temperatures, salinity, and drought conditions is *P. euphratica*, a unique poplar species commonly growing in hot and arid desert areas. These tolerances are explained by a pronounced epicuticular wax bloom to reflect light (Grünhofer et al. 2021b), small and succulent leaves to store water (Ottow et al. 2005; Xu et al. 2016), and further morphological adaptations to cope with excess sodium chloride (Chen and Polle 2010; Polle and Chen 2015). However, the observed high drought tolerance is only achieved by *P. euphratica* roots having constant contact with the groundwater table (Aishan et al. 2015; Chen et al. 2006; Zhou et al. 2010), which significantly decreases the occurrence of true water limitations.

Concerning root apoplastic barriers (Casparian bands and suberin lamellae), which are frequently discussed as means of a plant root to limit the uncontrolled loss of water and uptake of toxic ions (Grünhofer and Schreiber 2023), very little is known about perennial dicotyledonous trees (Brunner et al. 2015; Polle et al. 2019) and especially *Populus* as their model genus (Table 1). Here, further knowledge about tree root systems might be beneficial in selecting and breeding species and cultivars to optimize their cultivation on water-limited and saline soils unusable for food production (Polle and Chen 2015). Such information is available for several very well-studied annual monocotyledonous crop species, where the induction of suberin biosynthesis, an increase of root suberin contents, and an optional formation of an exodermis have frequently been identified to assist in conveying increased resistance against various abiotic stress conditions (Grünhofer et al. 2021c). Comparable and to a great degree coinciding data is also available for some dicotyledonous species such as, for example, *Arabidopsis* (Wang et al. 2020), castor bean (Schreiber et al. 2005), or cotton (Reinhardt and Rost 1995).

**Table 1:** The effect of different cultivation conditions and abiotic stress treatments on root apoplastic barriers of different poplar species. An exodermis is defined as a hypodermis exhibiting Casparian bands (Perumalla and Peterson 1986). ↑ = increase; ↓ = decrease; →  = no increase or decrease; +  = reported to be present; – = reported to be absent; *EN* Endodermis, *EX*  Exodermis, *w* Weeks, *d* Days; *GC* Gas chromatography, *Hydro* Hydroponic cultivation

Species	Plant age at harvest	Methods for apoplastic barrier analysis	Type of cultivation or stress	Suberization reaction	Exodermis formation	Reference
*P. maximowiczii* × *P. nigra*	11 w	Histochemistry	Hydro, platinum and lead stress		+, with picture but unsuitable staining method	Ballach and Wittig (1996)
*P. tremuloides*	12 w	Histochemistry	Sand, then Hydro		–, with picture	Wan and Zwiazek (2001)
*P. tremuloides*	16–18 w		Sand, water withdrawal		+, no picture	Siemens and Zwiazek (2003)
*P. tremuloides*	16–18 w		Sand, water withdrawal		+, no picture	Siemens and Zwiazek (2003)
*P. tremuloides*	8 w		Sand then Hydro		–, reference to Wan and Zwiazek (2001)	Voicu and Zwiazek (2004)
*P.* × *euramericana*	Rooting + 27 d	Histochemistry	Hydro, zinc stress	↑ in EN	–, with picture	Stoláriková et al. (2012)
*P. trichocarpa*	Mature trees	Histochemistry	Soil		+, with picture	Bagniewska-Zadworna et al. (2014)
*P. deltoides*	Rooting + 8 w	Histochemistry	Hydro, zinc stress	↓ in EN → in EX	+, no picture	Stoláriková-Vaculíková et al. (2015)
*P.* × *canescens*	5 w	Histochemistry and chemical analysis	Hydro, osmotic stress	(Slightly) ↑ in EN and EX	+, with picture	Grünhofer et al. (2022)
			Hydro, salinity stress	(Slightly) ↑ in EN and EX	+, with picture	

For monocotyledonous crops, it has been shown that the cultivation of roots in aeroponic or soil conditions (Krishnamurthy et al. 2009; Redjala et al. 2011; Tylová et al. 2017; Zimmermann et al. 2000), simulation of drought conditions by osmotic stress (Kreszies et al. 2019, 2020), treatment of roots with sodium chloride (Knipfer et al. 2020; Krishnamurthy et al. 2011), exogenous application of abscisic acid (ABA) to roots (Grünhofer et al. 2021c; Zeier 1998), or growth of roots in oxygen-deficient stagnant conditions (Kotula et al. 2009; Ranathunge et al. 2011a) all provoked significant endodermal and/or exodermal suberization reactions. An exodermis is defined as a hypodermis exhibiting Casparian bands (Perumalla and Peterson 1986), whose formation may, in high similarity to the chronological development of the endodermis (Krömer 1903), be followed by the deposition of suberin lamellae (Enstone and Peterson 1997; Hose et al. 2001). Although the endodermis and exodermis share some functional properties, the exodermis located at the outer cortex appears to be especially important in limiting the radial loss of oxygen from root tissue to the surrounding environment and inhibiting the diffusion of toxic solutes from the outside medium into the root (Enstone et al. 2003; Schreiber and Franke 2011).

The development of apoplastic root barriers of the dicotyledonous poplar species *Populus* × *canescens* (gray poplar) cultivated in hydroponic control conditions was investigated and thoroughly compared with the monocotyledonous barley species *Hordeum* *vulgare*, which both had been cultivated in the same laboratory using the same methods. In contrast to the highly stress-tolerant barley species exhibiting a functional zone of full endodermal suberization stretching from 50 to 100% relative root length (root tip = 0%, root base = 100%), roots of the stress-susceptible poplar species were characterized mostly by endodermal functional zones of no (0–27.5%) or only patchy (27.5–100%) suberization (Grünhofer et al. 2021a). It was very surprising to observe, that upon exposure of hydroponically cultivated *P.* × *canescens* roots to different intensities of osmotic (-0.4, -0.6, and -0.8 MPa induced with PEG8000) and salt (80, 120, and 160 mM NaCl) stress for 1 week, root suberization was not significantly or only weakly increased (Grünhofer et al. 2022). Although the formation of an exodermis could be observed in a small fraction of the roots of each treatment, the degree of suberization hardly changed at all. If major amounts of additional suberin were deposited in response to stress, this was only observed in the developing younger root tip region (0–27.5%), previously characterized by a functional zone of no suberization (Grünhofer et al. 2022). In contrast, highly comparable osmotic stress conditions led to a significantly increased suberization, especially in the zone of patchy suberization (25–50%), in barley (Kreszies et al. 2019).

Due to these observations, it was the aim of this study to investigate further cultivation conditions (aeroponics, in soil) and abiotic stress treatments (exogenous ABA, oxygen deficiency) of *P.* × *canescens* roots, known to significantly stimulate the apoplastic barrier formation in roots of other plant species.

## Materials and Methods

### Cultivation conditions and abiotic stress treatments

**Fig. 1:**
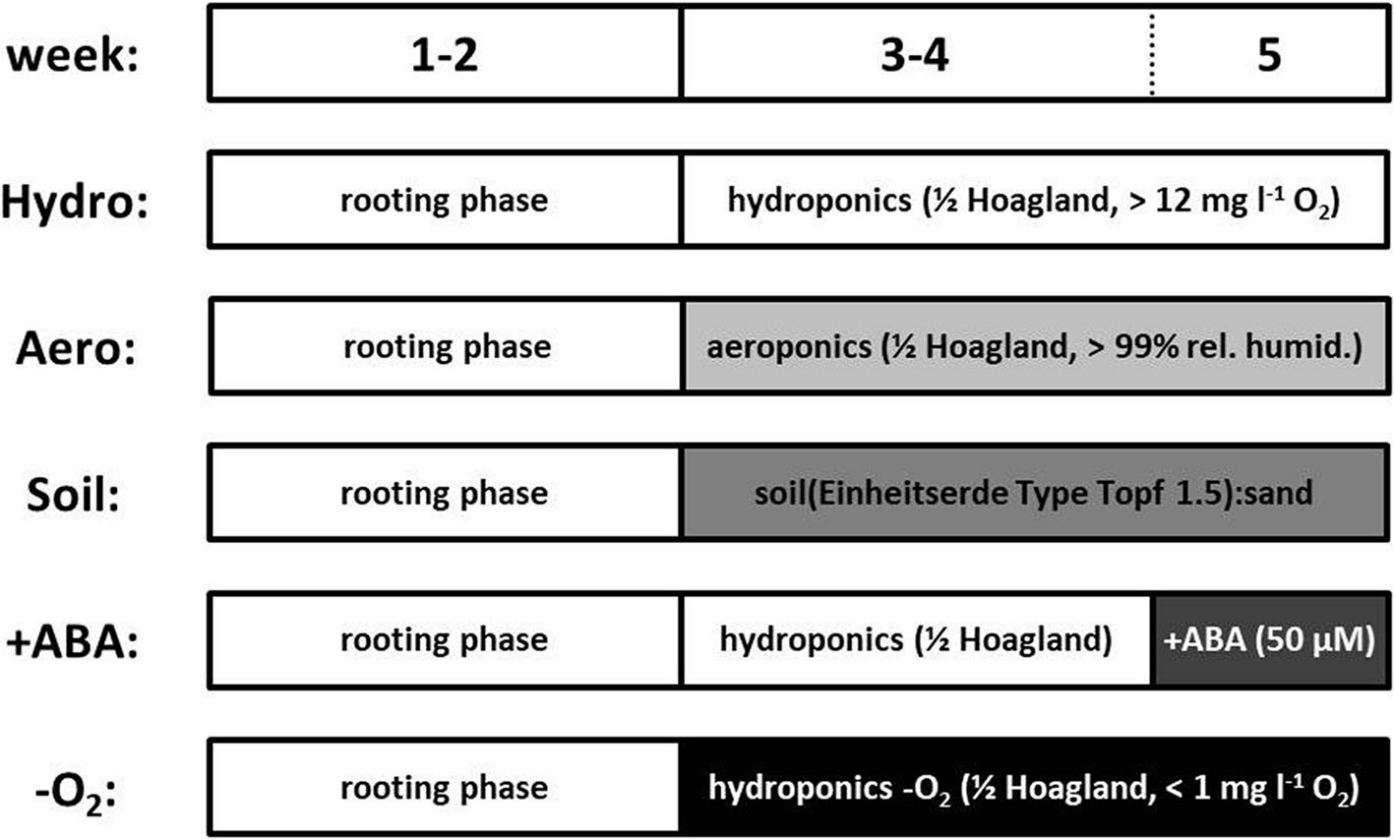
Experimental setup of all cultivation conditions and abiotic stress treatments. 14- to 18-week-old plants growing in soil were dissected into stem cuttings to initiate a 2-week-long rooting phase in stagnant tap water. After stem cuttings were rooted, they were assigned to the different cultivation conditions (‘Hydro’ = hydroponics, ‘Aero’ = aeroponics, ‘Soil’ = soil) or stress treatments (‘ +ABA’ = abscisic acid, ‘-O_2_’ = oxygen deficiency). Hydroponic cultivation served as control for all other experiments. Five stem cuttings were combined to yield one biological replicate and four biological replicates were grown per experiment. ½ Hoagland nutrient solution (Hoagland and Arnon 1950) was used in each experiment, either aerated in hydroponics, vaporized in aeroponics, as irrigation in soil cultivation, supplemented with ABA in the fifth week, or fumigated with gaseous nitrogen to achieve oxygen deficiency

The experiments of this study have been performed with *P.* × *canescens* (Aiton) Sm. clone ‘84 K’ (*P. alba* × *P. tremula* var. *glandulosa*) (Qiu et al. 2019). This poplar species is characterized by a low tolerance against salinity (Bolu and Polle 2004) and osmotic stress (Grünhofer et al. 2022), is susceptible to oxidative stress (Strohm et al. 1999), but can cope well with increased concentrations of phosphorus (Kavka and Polle 2016) and flooding exposure (Kreuzwieser et al. 2009). Poplar plants were propagated and grown in axenic tissue culture for 6 to 8 weeks, followed by transplantation into soil with subsequent acclimatization for 2 weeks, and a final cultivation period in soil of a further 6 to 8 weeks after acclimatization. The climate chamber was set to long-day conditions with a 16 h/8 h light/dark period, 21 °C/19 °C mean temperature, 50%/67% mean relative humidity, and a light intensity of 100 µmol m^−2^ s^−1^ during illumination. After poplar plants had reached a total age of 14 to 18 weeks, they were dissected into stem cuttings and rooted (resulting exclusively in stem-borne adventitious roots; Bellini et al. 2014) in stagnant tap water for 2 weeks before being assigned to the respective cultivation conditions or abiotic stress treatments (Fig. 1). Further details about hydroponic *P.* × *canescens* cultivation as well as histochemical and chemical analysis of poplar root suberin can be found in Grünhofer et al. (2021a).

Hydroponic cultivation (‘Hydro’, also serving as a control for all cultivation conditions and abiotic stress treatments and executed in batches at different points in time) consisted of 3-week-long plant growth after rooting in pots filled with aerated ½ Hoagland nutrient solution (Hoagland and Arnon 1950). Aeration of the solution resulted in oxygen levels of > 12 mg l^−1^ (PCE-PHD 1, PCE Instruments, Germany) and the water potential was previously measured (freezing point osmometer, OSMOMAT 030, gonotec, Germany) to be -0.037 MPa (Grünhofer et al. 2021a). The nutrient solution was renewed once a week.

Aeroponic cultivation (‘Aero’) was performed with ultrasonic humidifiers (‘Mist Maker Humidifer’, 1 membrane, 573 ml h^−1^ capacity, LED Grower, Czech Republic) located inside the pots and submerged in ½ Hoagland solution for 3 weeks after rooting. The humidifiers ensured a relative humidity of > 99% (PCE-WM 1, PCE Instruments, Germany). However, relative humidity can be converted into water potential and values of, for example, 100, 99.9, and 99.8% correspond to 0, -0.14, and -0.27 MPa, respectively (Grünhofer and Schreiber 2023). Due to the lower amounts of nutrient solution used, it was renewed every third day.

Soil cultivation (‘Soil’) was conducted by planting rooted stem cuttings into pots filled with a 1:1 soil (Einheitserde Classic Type Topf 1.5, Einheitserde Werksverband e.V., Germany) and sand (quartz sand) mixture and growing them for further 3 weeks after rooting. The soil water potential was kept at -0.16 ± 0.11 MPa (WP4C Dewpoint PotentiaMeter, Decagon Devices, USA) by keeping the pots in a tray constantly filled with ½ Hoagland solution.

Abscisic acid stress treatment (‘ +ABA’) was executed by cultivating rooted stem cuttings in unmodified ½ Hoagland solution for 2 weeks, before adding 50 µM abscisic acid (ABA, 2-*cis*,4-*trans*-abscisic acid, Sigma Aldrich, Germany) into the nutrient solution at the beginning of the final fifth week. Supplementation with ABA did not affect the water potential of the medium. The nutrient solution was renewed once a week.

Oxygen deficiency stress treatment (‘-O_2_’) was induced right after the rooting of stem cuttings in stagnant tap water. Rooted cuttings were cultivated in ½ Hoagland solution for 3 weeks, but this time the nutrient solution was constantly fumigated with gaseous nitrogen instead of air. This bubbling with N_2_ resulted in oxygen levels of < 1 mg l^−1^ without affecting the water potential of the medium. The nutrient solution was also renewed once a week. Oxygen deficiency, the treatment focused on in our study, and deoxygenated stagnant conditions (‘Stag’), the treatment focused on in some references mentioned in the introduction and discussion (e.g., Ranathunge et al. 2011a or Kotula et al. 2009), are similar but not exactly the same due to increased diffusive boundary layers as a consequence of no constant fumigation and thus potentially higher ethylene accumulation during stagnation (Colmer et al. 1998; Colmer 2003). Ethylene, in turn, can be an important factor in stimulating inducible aerenchyma formation which provides a pathway of low resistance for the movement of gases from the shoot to the root (Pedersen et al. 2020). This is why a histological comparison of aerated hydroponics, oxygen deficiency, stagnation for 3 weeks after rooting (‘Stag long’), and stagnation for 1 week in the final fifth week (‘Stag short’) was performed before choosing one of these stress treatments for further detailed analyses (Fig. S1a). To prepare the stagnant solution, 0.1% (w/v) agar (Sigma Aldrich, Germany) was dissolved in water and boiled. After cooling, thus avoiding precipitation due to high temperatures, macro- and micronutrients were added exactly as for the ½ Hoagland solution of the hydroponic cultivation. To finally reduce the oxygen concentration in the stagnant medium below 1 mg l^−1^, the solution was fumigated once with gaseous nitrogen for 20 min (Kotula et al. 2009). For the long stagnation treatment, the nutrient solution was renewed weekly. Since no histochemically observable differences could be identified in the suberin lamellae deposition (Fig. S1b) or Casparian band development (Fig. S1c) in the even more stressful stagnant conditions when compared to the oxygen deficiency treatment, it was decided to focus the following analyses (plant physiological measurements, chemical analysis of roots) on the -O_2_ treatment due to more feasible handling of roots and certainly no adverse effect of supplemented agar on the water potential of the nutrient solution.

In every experiment, five stem cuttings were combined to yield one biological replicate. In the case of Hydro, Aero, +ABA, and -O_2_ the five stem cuttings of a biological replicate were cultivated in a shared larger pot (diameter of 15 cm, height of 20 cm, volume of 3.5 l), whereas for Soil each stem cutting was growing in an individual smaller pot (lengths of 10 × 10 × 10 cm, volume of 1 l) to avoid tangling of roots.

### Plant physiology

To quantify the impacts of different cultivation (Hydro, Aero, Soil) and abiotic stress (+ABA, -O_2_) conditions (hereafter comprehensively termed ‘experiment’) on plant growth, the stomatal conductances of leaves (measured between 14 and 16 o’clock of a given day; LI-600 Leaf Porometer, Li-Cor, USA), osmotic potentials of leaves and roots (freezing point osmometer), and the mean root length (the length of each individual adventitious root was measured with a ruler) were determined at the day of harvest after 5 weeks of plant cultivation in total (Fig. 1). In addition, the stomatal conductances of leaves of the +ABA treatment and its hydroponic control were measured shortly before stress application at 4 weeks, as well as 10 min, 1 day, 2 days, and 6 days after stress application, and at the end (5 weeks) of the cultivation phase. This was done, because the ABA application was the only treatment performed for a shorter time period of only 1 week (Fig. 1) and ABA is known for its stomatal closure inducing effects. Thus, tightly measuring the stomatal behavior allowed monitoring the effectiveness of the ABA treatment even if no suberization induction could be observed.

### Histochemistry

Only adventitious roots that exhibited root lengths close (± 3 cm) to the calculated mean of an experiment were selected for histochemical analysis, and for each experiment at least 6 roots were investigated. Fixated roots (3.7% v/v formaldehyde, 10 mM Na_2_HPO_4_, 137 mM NaCl, 2.7 mM KCl, pH 7.4) were divided into 1 cm segments before being cut into 30 µM thick cross-sections with a cryostat microtome (Microm HM 500 M, Microm International GmbH, Germany). Prepared cross-sections of the whole root diameter (comprising both endodermis and hypodermis) were then stained with 0.1% (w/v) berberine hemi-sulphate and 0.5% (w/v) aniline blue (Brundrett et al. 1988) to detect Casparian bands or with 0.01% (w/v) fluorol yellow 088 (Brundrett et al. 1991) to detect suberin lamellae development. Epifluorescence microscopy was carried out using an ultraviolet (UV) filter set (excitation filter BP 365, dichroic mirror FT 395, barrier filter LP 397; Zeiss, Germany). Representative photographs were taken with a Canon EOS 600D camera (Canon Inc., Japan) and edited using ImageJ (Abramoff et al. 2004). Due to brightness adjustments, the color intensity does only reflect the Casparian band and suberin lamellae localization, but not quantitative amounts. To ensure high comparability to previously generated data, all investigated root segments were expressed as relative lengths of the whole root. Here, 0% relative root length represents the root tip, and 100% relative root length represents the root base (Kreszies et al. 2019). Based on 5-week-long poplar cultivation in hydroponic cultivation conditions, two functional developmental zones were defined. Zone A (0–27.5%) showed no signs of endodermal suberization, whereas Zone B (27.5–100%) was characterized by a constantly increasing patchy suberization of the endodermis towards the root base (Grünhofer et al. 2021a).

### Chemical analysis

The same developmental zones (Zone A 0–27.5%, Zone B 27.5–100%) of roots close to the calculated mean of an experiment were also chemically analyzed by gas chromatography (GC) coupled to flame ionization detection (GC-FID) or mass spectrometry (GC–MS). After the removal of lateral roots from the fixated main root, 10 to 20 adventitious roots were dissected into the two developmental zones and pooled to yield one biological replicate for suberin analysis. To remove all extractable primary cell wall components and membrane lipids, the pooled root zones were treated with 0.5% (w/v) cellulase and 0.5% (w/v) pectinase for 2 weeks, borate buffer for 1 day, and 1:1 (v/v) chloroform:methanol for further 2 weeks (Zeier and Schreiber 1997). The enzyme and organic solvent solutions were renewed every third day. After drying of the remaining polymerized and non-extractable cell wall components on polytetrafluoroethylene (PTFE), the dry weight of samples could be measured. Depolymerization and transesterification were carried out using BF_3_-methanol (Zeier and Schreiber 1998) and the released suberin monomers were spiked with 10 µg of internal standard (Dotriacontane, Fluka, Germany). After repeated extraction of monomers with chloroform, the sample volume was reduced at 60 °C under a gentle stream of N_2_. To mask reactive hydroxyl groups of alcohols and bi-functional acids with trimethylsilyl (TMS) protective groups, the sample was derivatized with 20 µl pyridine (Sigma Aldrich, Germany) and 20 µl BSTFA (N,O-Bis(trimethylsilyl)trifluoroacetamide, Macherey–Nagel, Germany). Finally, splitless GC-FID (6890N, Agilent Technologies, USA) and GC–MS (GC–MS: 7890B-5977A, Agilent Technology, USA) analysis was performed with the temperature program published by Delude et al. (2017) (50 °C for 1 min, a temperature increase of 25 °C min^−1^ up to 200 °C, 1 min at 200 °C, 10 °C min^−1^ up to 320 °C, and final hold for 8 min at 320 °C). The identified and quantified suberin monomer amounts were related to the endodermal surface area of each zone, which was calculated based on the cross-sections of roots that were used for histochemical studies and a truncated cone shape of roots (Grünhofer et al. 2021a).

### Statistical analysis

Each experiment (Hydro, Aero, Soil, +ABA, -O_2_) and measurement (e.g., stomatal conductances, osmotic potentials, etc.) was executed with at least 3 biological replicates. Generated data was analyzed and visualized with OriginPro 20 (OriginLab Corporation, USA). The normal distribution of data was evaluated with the Shapiro–Wilk test, whereas significant differences were examined using a two-sample *t*-test or one-way ANOVA with Fisher’s LSD post hoc test. Significant differences (*P* ≤ 0.05) are indicated by differential letters. Means with standard deviations are shown in all graphs.

## Results

### Plant physiology

**Fig. 2:**
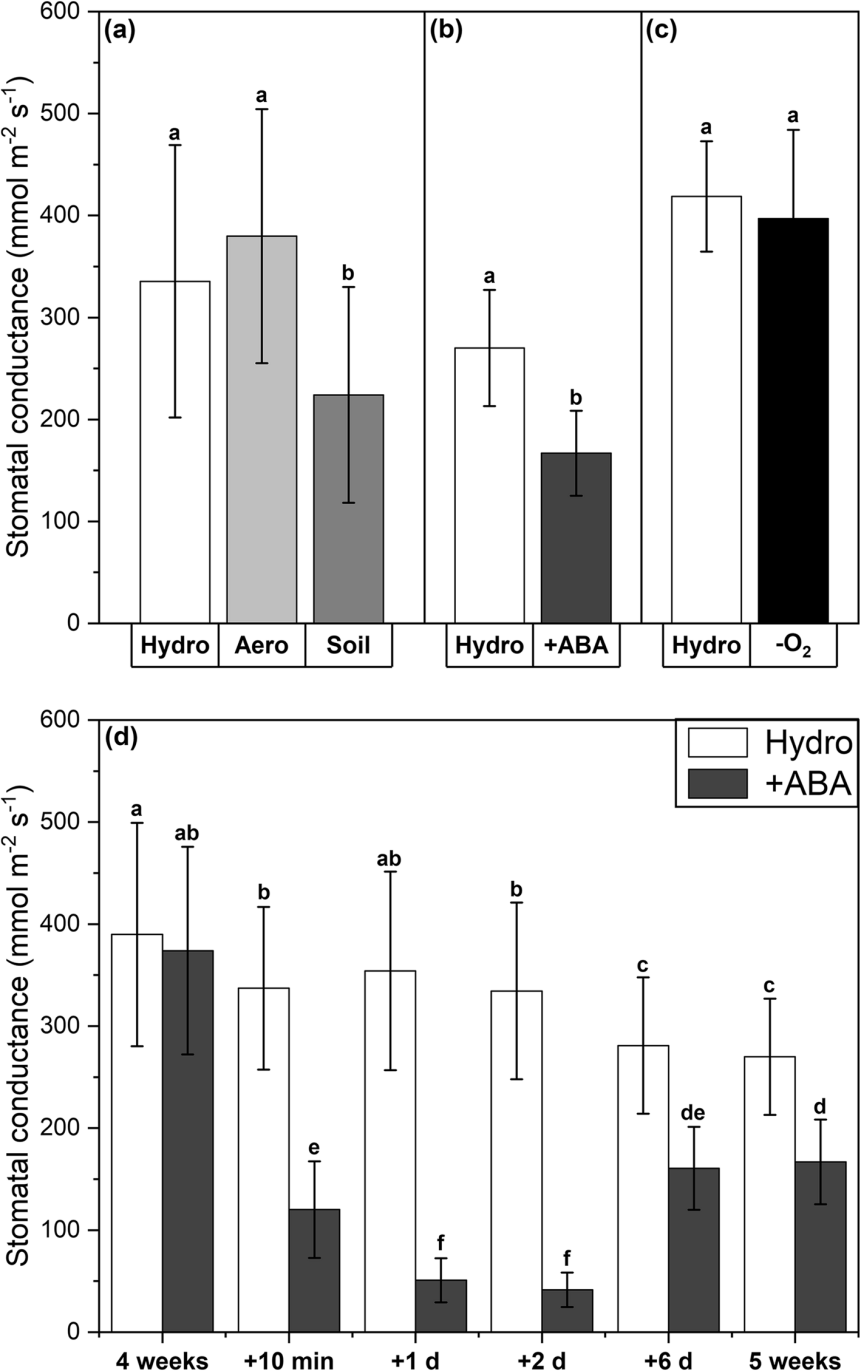
Stomatal conductances of leaves after plant growth in different cultivation conditions (**a**) and abiotic stress treatments (**b**-**d**). Experiments included hydroponic (‘Hydro’), aeroponic (‘Aero’), and soil (‘Soil’) cultivation as well as abscisic acid (‘ +ABA’) and oxygen deficiency (‘-O_2_’) stress treatment. Hydroponic cultivation served as control for all other experiments. (**a**-**c**) Measurements were carried out at the day of harvest after 5 weeks of plant cultivation. (**d**) Measurements were carried out shortly before stress application (4 weeks) as well as 10 min, 1 day, 2 days, and 6 days after stress application, and at the end (5 weeks) of the cultivation phase. Means with standard deviations are shown. *n* = 5–20 leaves. Differential letters indicate significant differences at *P* < 0.05

At plant harvest after 5 weeks of cultivation, the stomatal conductances of leaves grown in the soil cultivation (Fig. 2a) and +ABA treatment (Fig. 2b) were significantly reduced by 33% (*P* = 0.015) and 38% (*P* < 0.001), respectively, compared to the corresponding control. In contrast, the aeroponic cultivation (Fig. 2a) and -O_2_ treatment (Fig. 2c) did not affect the stomatal conductance of leaves. To monitor the effectiveness of the ABA application and investigate the time needed for ABA to induce stomatal closure, a series of stomatal conductance measurements was executed (Fig. 2d). Already 10 min after ABA application, stomatal conductances of leaves decreased significantly (*P* < 0.001), and the lowest values were reached after 1 day.

**Fig. 3:**
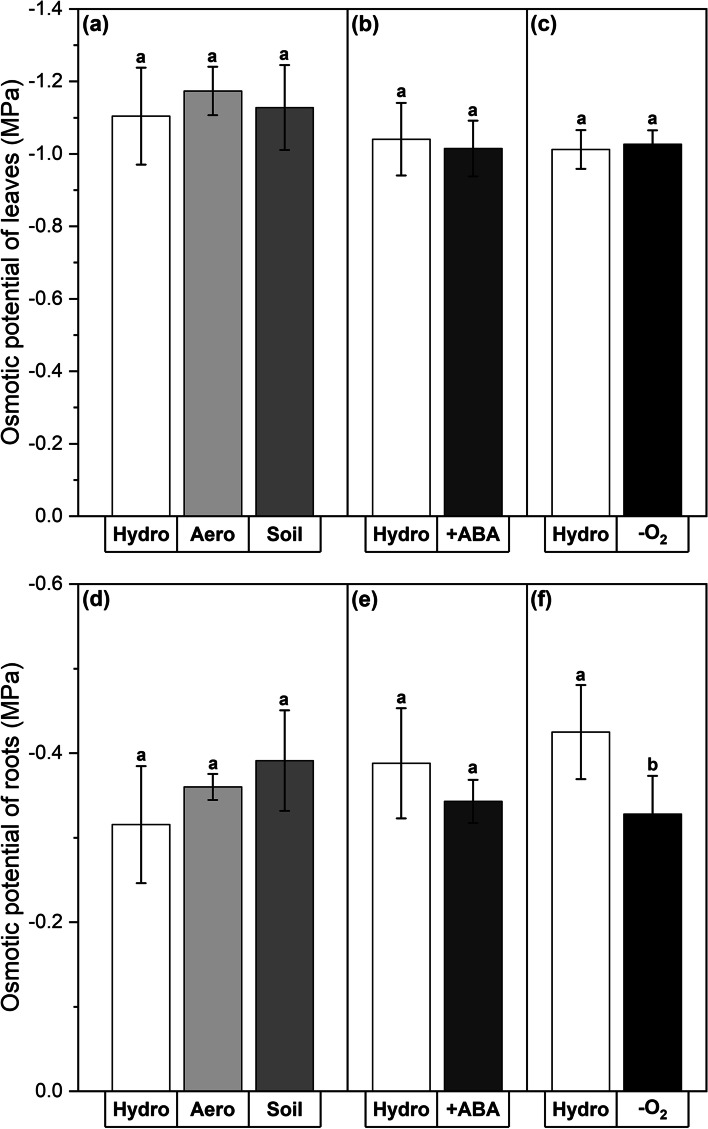
Osmotic potentials of leaves (**a**-**c**) and roots (**d**-**f**) after plant growth in different cultivation conditions and abiotic stress treatments. Experiments included hydroponic (‘Hydro’), aeroponic (‘Aero’), and soil (‘Soil’) cultivation as well as abscisic acid (‘ +ABA’) and oxygen deficiency (‘-O_2_’) stress treatment. Hydroponic cultivation served as control for all other experiments. All measurements were carried out at the day of harvest after 5 weeks of plant cultivation. Means with standard deviations are shown. *n* = 4–10 leaves or roots. Differential letters indicate significant differences at *P* < 0.05

The different cultivation conditions or abiotic stress treatments did not affect the osmotic potentials of leaves (Fig. 3a-c) or roots (Fig. 3d-f), with the single exception of a significant (*P* = 0.016) but only minor increase of the osmotic potential of roots grown in the -O_2_ treatment (Fig. 3f).

**Fig. 4:**
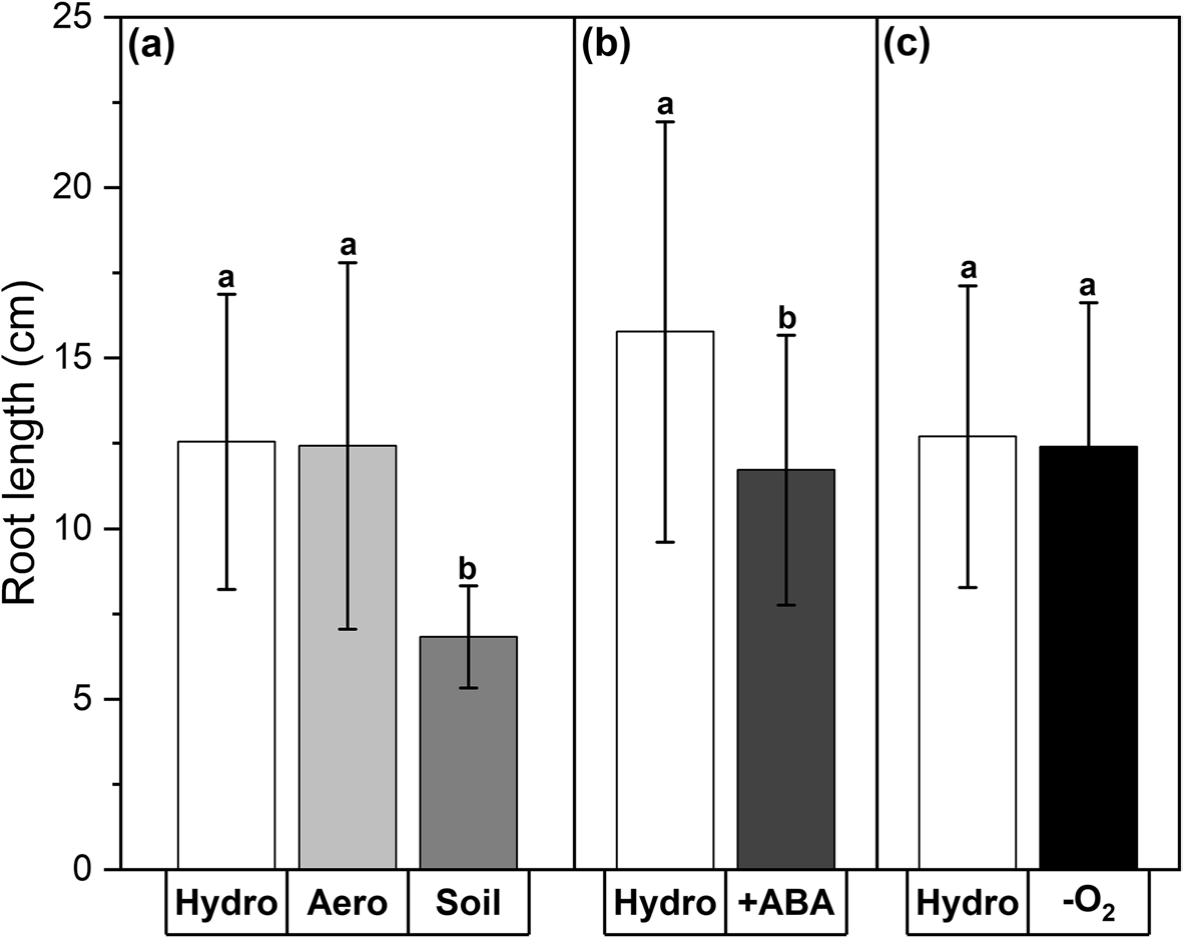
Adventitious root lengths after plant growth in different cultivation conditions (**a**) and abiotic stress treatments (**b**,**c**). Experiments included hydroponic (‘Hydro’), aeroponic (‘Aero’), and soil (‘Soil’) cultivation as well as abscisic acid (‘ +ABA’) and oxygen deficiency (‘-O_2_’) stress treatment. Hydroponic cultivation served as control for all other experiments. All measurements were carried out at the day of harvest after 5 weeks of plant cultivation. Only roots > 5 cm were considered. Means with standard deviations are shown. *n* = 48–167 individual roots. Differential letters indicate significant differences at *P* < 0.05

Similar to the stomatal conductances of leaves (Fig. 2), adventitious root lengths (only roots > 5 cm were considered) grown in the soil cultivation (Fig. 4a) and +ABA treatment (Fig. 4b) were significantly reduced by 46% (*P* < 0.001) and 26% (*P* < 0.001), respectively. But aeroponics cultivation (Fig. 4a) or -O_2_ treatment (Fig. 4c) did not affect the root length development.

### Histochemistry

**Fig. 5:**
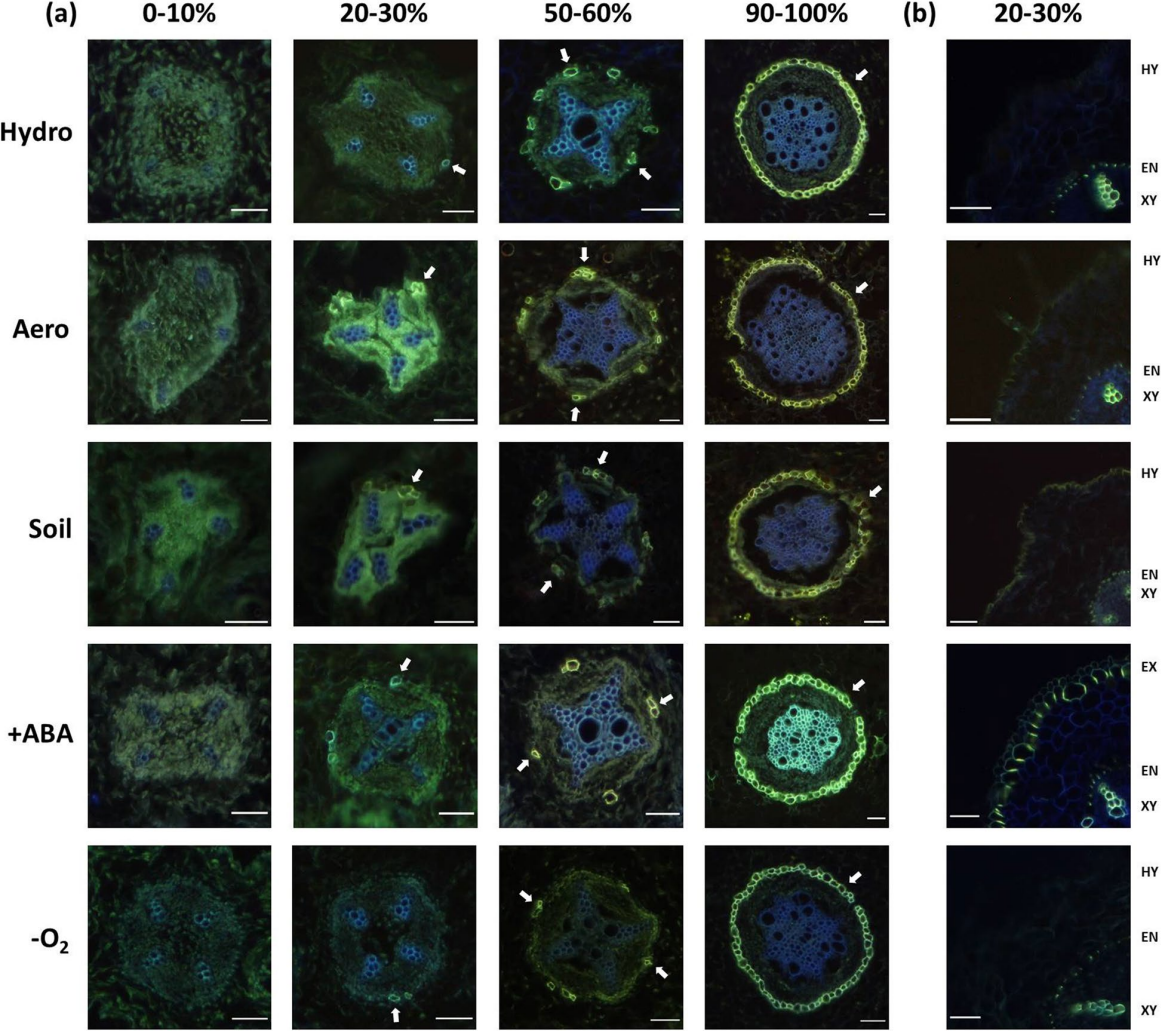
Suberin lamellae (**a**) and Casparian band (**b**) development after plant growth in different cultivation conditions and abiotic stress treatments. Experiments included hydroponic (‘Hydro’), aeroponic (‘Aero’), and soil (‘Soil’) cultivation as well as abscisic acid (‘ +ABA’) and oxygen deficiency (‘-O_2_’) stress treatment. Hydroponic cultivation served as control for all other experiments. The relative distances from the root apex (0%) to the root base (100%) are given. (**a**) Suberin lamellae deposition was visualized using fluorol yellow 088 applied to the whole root cross-section. However, since no suberin staining was ever achieved in the hypodermis of roots of any experiment, the figure focuses on the endodermal suberin lamellae development. Arrows indicate the onset of suberization (20–30%), transition into patchy suberization (50–60%), and almost full suberization (90–100%) of the endodermis. Scale bars = 100 µm. (**b**) Casparian band development was visualized using berberine-aniline blue applied to the whole root cross-section. An early exodermis formation (hypodermis with Casparian bands) was observed only in a small fraction of roots of the +ABA treatment and only in the apical 20–30% relative root length (see EX indicator on the right; a representative photograph is shown here) but not in roots of any other experiment (see HY indicator on the right). The endodermal Casparian bands of roots of all experiments developed highly comparable to that of the hydroponic control (see EN indicator on the right). No development of an aerenchyma within the root cortex has been observed in any cultivation condition or stress treatment. Scale bars = 100 µm. *n* = 6 or more roots. *EN* Endodermis, *EX* Exodermis, *HY* Hypodermis, *XY *Xylem

No differences were observed for the endodermal Casparian band and endodermal suberin lamellae development (Fig. 5) along the root. In all cultivation conditions and stress treatments, endodermal Casparian bands were already fully developed at 10–20% relative root length (no data shown). Endodermal suberization started at 20–30% relative distance and continued in a patchy manner to reach a full suberization, if at all, in the basal 90–100% relative root length (Fig. 5a). Also, the hypodermal development was affected only weakly. Neither hypodermal Casparian bands (Fig. 5b) nor hypodermal suberin lamellae deposition (no data shown) could be observed, with the single minor exception being the +ABA treatment. Here, a small fraction of investigated roots showed signs of an early exodermis development indicated by the apical formation of hypodermal Casparian bands at 20–30% relative root length (Fig. 5b), but yet no observable suberin lamellae deposition (no data shown). Also, no development of an aerenchyma within the root cortex has been observed in any cultivation condition or stress treatment (no data shown).

### Chemical analysis

**Fig. 6:**
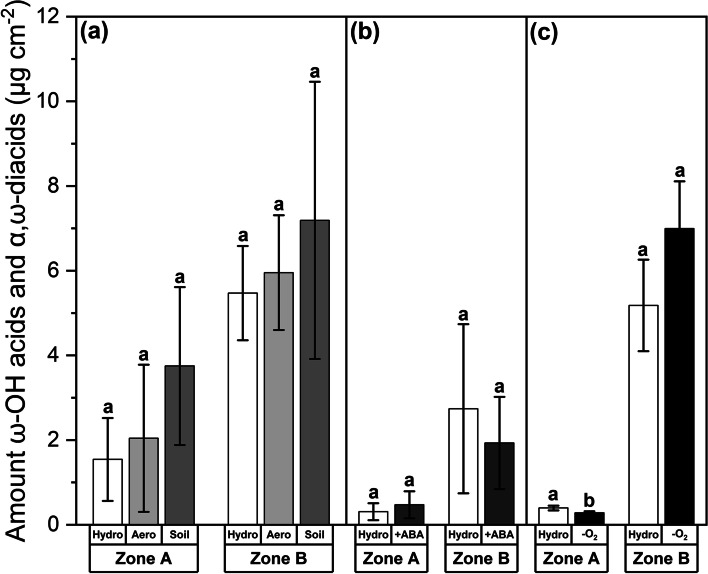
Amounts of the aliphatic suberin diagnostic functional groups ω-hydroxy acids (ω-OH acids) and α,ω-dicarboxylic acids (α,ω-diacids) after plant growth in different cultivation conditions (**a**) and abiotic stress treatments (**b**,**c**). Experiments included hydroponic (‘Hydro’), aeroponic (‘Aero’), and soil (‘Soil’) cultivation as well as abscisic acid (‘ +ABA’) and oxygen deficiency (‘-O_2_’) stress treatment. Hydroponic cultivation served as control for all other experiments. Roots were divided into the functional Zone A (0–27.5% relative root length) and Zone B (27.5–100% relative root length). Amounts were related to the endodermal surface area. Means with standard deviations are shown. *n* = 4 biological replicates. Differential letters indicate significant differences at *P* < 0.05

The chemical analysis of root aliphatic suberin (Fig. 6) corresponds well to the histochemical observations (Fig. 5). No significant increases or decreases of the suberin diagnostic functional groups ω-hydroxy acids (ω-OH acids) and α,ω-dicarboxylic acids (α,ω-diacids) could be identified in either the apical Zone A or basal Zone B in response to any cultivation condition (Fig. 6a) or stress treatment (Fig. 6b,c). The single exception here is represented by a significant (*P* = 0.029) yet minor decrease of suberin amounts in Zone A of the -O_2_ treatment (Fig. 6c). Even when analyzing the distribution of all functional groups (Fig. S2) or chain lengths (Fig. S3) of aliphatic root suberin, no significant differences in absolute amounts (Fig. S2a, Fig. S3a) and only slight alterations in relative amounts (Fig. S2b, Fig. S3b) could be identified. An exception to this observation is the significantly (*P* = 0.011) increased combined absolute aliphatic suberin amount in Zone A of roots originating from the soil cultivation, which is essentially caused by a high deposition of primary C16 and C18 fatty acids (Fig. S2a, Fig. S3a) that are no part of the previously mentioned suberin diagnostic functional groups.

## Discussion

Since it was ensured that none of the imposed cultivation conditions or stress treatments represented considerably water-deficient environments (measured water potential values were never below -0.3 MPa), secondary effects of osmotic stress can be ruled out.

While no significant or biologically relevant effects of all experiments on the osmotic potentials of leaves or roots were found (Fig. 3), the measurements of leaf stomatal conductances (Fig. 2) and root lengths (Fig. 4) revealed a significant influence of soil cultivation and +ABA treatment on the development of poplar plants. Leaf transpiration was reduced and root growth was delayed in both cases. Indeed, the effects of ABA on leaf transpiration were visible already 10 min after application (after 4 weeks of hydroponic control cultivation) and lasted throughout the whole fifth week (Fig. 2d). The negative effect of ABA on poplar root length development is in line with what has been described before for other monocotyledonous species (Grünhofer et al. 2021c). However, the reductive impact of soil cultivation on poplar root lengths was surprising since previous studies with monocotyledonous maize found the root lengths to increase significantly (Redjala et al. 2011; Tylová et al. 2017). Together with the also decreased stomatal conductance during soil growth, it appears that soil cultivation represents a significant initial stress factor for poplar growth. This might be due to the suddenly increased exposure to beneficial and pathogenic microbiota living in the soil and could be overcome as soon as the plant has acclimatized to the newly imposed conditions, for example by the activation of symbiosis or disease resistance mechanisms, respectively (Tsai et al. 2006; Tuskan et al. 2006). Responses of poplar roots to aeroponics and oxygen deficiency were also unexpected since root lengths did not change in both experiments whereas it was reported for monocotyledonous maize and rice that root lengths increased in response to aeroponic cultivation (Miyamoto et al. 2001; Redjala et al. 2011) and decreased in response to stagnant -O_2_ treatment (Kotula et al. 2009; Ranathunge et al. 2011a; Shiono et al. 2014). In this case, the fine difference between oxygen deficiency with N_2_ fumigation and deoxygenated stagnation might be of great importance, because contrastingly the root lengths (although not precisely quantified because it was decided to focus on the oxygen deficiency treatment) of the long and short stagnant treatment were found to be slightly and considerably decreased, respectively (no data shown). Nonetheless, *P.* × *canescens* is well known to thrive in habitats that are characterized by frequent flooding (van Loo et al. 2008), and thus a specialized adaptation to this floodplain forest environment (Netzer et al. 2018) including a general flooding tolerance (Kreuzwieser et al. 2009) can certainly be expected.

In parallel, no histochemically observable alterations of endodermal or hypodermal suberin lamellae development in any cultivation condition or stress treatment were found (Fig. 5a), which was also matching with the chemical analysis (Fig. 6, S2, S3). Thus, responses of poplar roots are considerably different from monocotyledonous crop species where it was reported that root suberin amounts increased upon aeroponic (Zimmermann et al. 2000) and soil (Krishnamurthy et al. 2009; Redjala et al. 2011; Tylová et al. 2017) cultivation as well as exogenous ABA application (Grünhofer et al. 2021c; Zeier 1998) and oxygen deficiency (Kotula et al. 2009; Ranathunge et al. 2011a).

While the sporadic observation of an exodermis formation after +ABA treatment (Fig. 5a) confirmed potentially apoplastic barrier inductive effects of ABA also observed previously (Barberon 2017), the lacking hypodermal modification after -O_2_ treatment in this study was surprising. It is very well known that oxygen deficiency induces or enhances the formation of an exodermis to limit radial oxygen loss to the surrounding stagnant medium (Kotula et al. 2009; Ranathunge et al. 2011a). This often coincides with a constitutive or induced aerenchyma development to facilitate the diffusion of oxygen to the growing root tip (Pedersen et al. 2020). Many poplar species are known to naturally grow in sporadically flooded and thus potentially oxygen-deficient environments (Eckenwalder 1996; Isebrands and Richardson 2014) and several studies with different poplar species had reported the presence of an exodermis previously (Table 1).

The observed inability of *P.* × *canescens* to form an aerenchyma or an exodermis upon oxygen deficiency indicates that different strategies to deal with reduced levels of O_2_ are needed in *P.* × *canescens*. It was observed with two *P. deltoides* × *P. simonii* clones differing in flood tolerance (Peng et al. 2017) that the flood-tolerant clone exhibited a smaller degree of aerenchyma formation when compared to the flood-susceptible clone. This was attributed to a concomitant increase in radial oxygen loss and increasingly disorganized root anatomy as a consequence of aerenchyma development. Unfortunately, the formation of hypodermal Casparian bands (exodermis formation) was not investigated in this study (Peng et al. 2017). The entirely missing formation of an aerenchyma in *P.* × *canescens* roots has also been reported before (Kreuzwieser et al. 2009). Here, oxygen deficiency was applied for only 1 week and the authors argued that at least 2 to 3 weeks are needed to induce an aerenchyma (Kreuzwieser et al. 2009). However, the prolonged -O_2_ treatment performed in this study (2 weeks of stagnant rooting + 3 weeks of controlled -O_2_ conditions) was also not sufficient to induce an exodermis or aerenchyma formation in *P.* × *canescens* roots. Obviously, in *P.* × *canescens* roots different mechanisms seem sufficient to cope with a limited oxygen supply, such as an enhancement of carbohydrate availability due to starch and sucrose degradation, which ultimately permits a switch from mitochondrial respiration to alcoholic fermentation in flooded root tissue (Kreuzwieser et al. 2009).

Stress-induced apoplastic barrier formation reactions described here with the dicotyledonous *P.* × *canescens* are considerably different from those known for monocotyledonous crop species (Grünhofer et al. 2021c). These differences include, for example, heterogeneous root length alterations in reaction to aeroponic cultivation, soil cultivation, and -O_2_ treatment as well as a remarkably low endodermal suberization or almost absent exodermis formation reaction in *P.* × *canescens* in response to all cultivation conditions and abiotic stress treatments. This indicates that pronounced differences in abiotic stress reactions must exist between annual monocotyledonous crop species and perennial dicotyledonous trees.

Three hypotheses could explain these differences in *P.* × *canescens* root responses to abiotic stress: (i) secondary growth of dicotyledonous roots significantly alters the root architecture since the whole cortex including the endodermis will functionally be replaced by a developing periderm (Esau 1977); (ii) an overall slower reaction pattern in response to abiotic stress, since tree root systems are very expansive and stress signals might only occur locally, allowing the tree to outlast certain environmental changes without functional modifications; and (iii) increased importance of young and developing lateral roots, non-woody fine roots, and root tips, which are most significantly contributing to the water and nutrient uptake (Gambetta et al. 2013). Root tips, and not older root zones, have also been identified in a previous study to be most reactive in response to osmotic and salt stress in *P.* × *canescens* (Grünhofer et al. 2022).

The results presented here and recent observations (Grünhofer et al. 2022) allow concluding that an apoplastic barrier formation in *P.* × canescens primary roots in response to various abiotic stress factors (osmotic and salt stress, ABA application, and oxygen deficiency) is largely missing. Genetically enhancing root apoplastic barrier biosynthesis key genes (Ranathunge et al. 2011b; Vishwanath et al. 2015) could aid in the optimization or acceleration of these stress reaction patterns observed in other species and might be beneficial in the generation of more stress-tolerant poplar cultivars in future. Of course, it needs to be considered that the findings of this study are only representative of young primary roots and older roots with a significantly developed periderm may be expected to exhibit different reaction patterns.

## Supplementary Information

**Additional file 1: Fig. S1. **Experimental setup (a) and histochemical results (b, c) of the oxygen deficiency and stagnant conditions comparison. **Fig. S2. **Absolute (a) and relative (b) amounts of the aliphatic suberin functional groups. **Fig. S3. **Absolute (a) and relative (b) amounts of the aliphatic suberin carbon chain length distribution.

## Data Availability

The datasets generated during and/or analyzed during the current study are available from the corresponding author on reasonable request.

## Abbreviations

Hydro: Hydroponic cultivation
Aero: Aeroponic cultivation
Soil: Soil cultivation
+ABA: Abscisic acid stress
-O_2_: Oxygen deficiency stress
Stag: Stagnant conditions
EN: Endodermis
EX: Exodermis
HY: Hypodermis
XY: Xylem
