# Supplementary material for: Apoplastic barriers of Populus × canescens roots in reaction to different cultivation conditions and abiotic stress treatments
Source: Stress Biol. 2023 Jul 21;3(1):24. doi: 10.1007/s44154-023-00103-3 (PMC10441858; doi:10.1007/s44154-023-00103-3)
Supplement: Supplementary file 1 — Additional file 1: Fig. S1. Experimental setup (a) and histochemical results (b, c) of the oxygen deficiency and stagnant conditions comparison. Fig. S2. Absolute (a) and relative (b) amounts of the aliphatic suberin functional groups. Fig. S3. Absolute (a) and relative (b) amounts of the aliphatic suberin carbon chain length distribution. [file 44154_2023_103_MOESM1_ESM.docx]

Title: Apoplastic barriers of *Populus* × *canescens* roots in reaction to different cultivation conditions and abiotic stress treatments

Journal: Stress Biology

Authors: Paul Grünhofer^1,*^, Ines Heimerich^1^, Lena Herzig^1^, Svenja Pohl^1^, Lukas Schreiber^1^

Contact information:

^1^Department of Ecophysiology, Institute of Cellular and Molecular Botany, University of Bonn, Kirschallee 1, 53115 Bonn, Germany

^*^Author for correspondence: Paul Grünhofer

Email: p.gruenhofer@uni-bonn.de


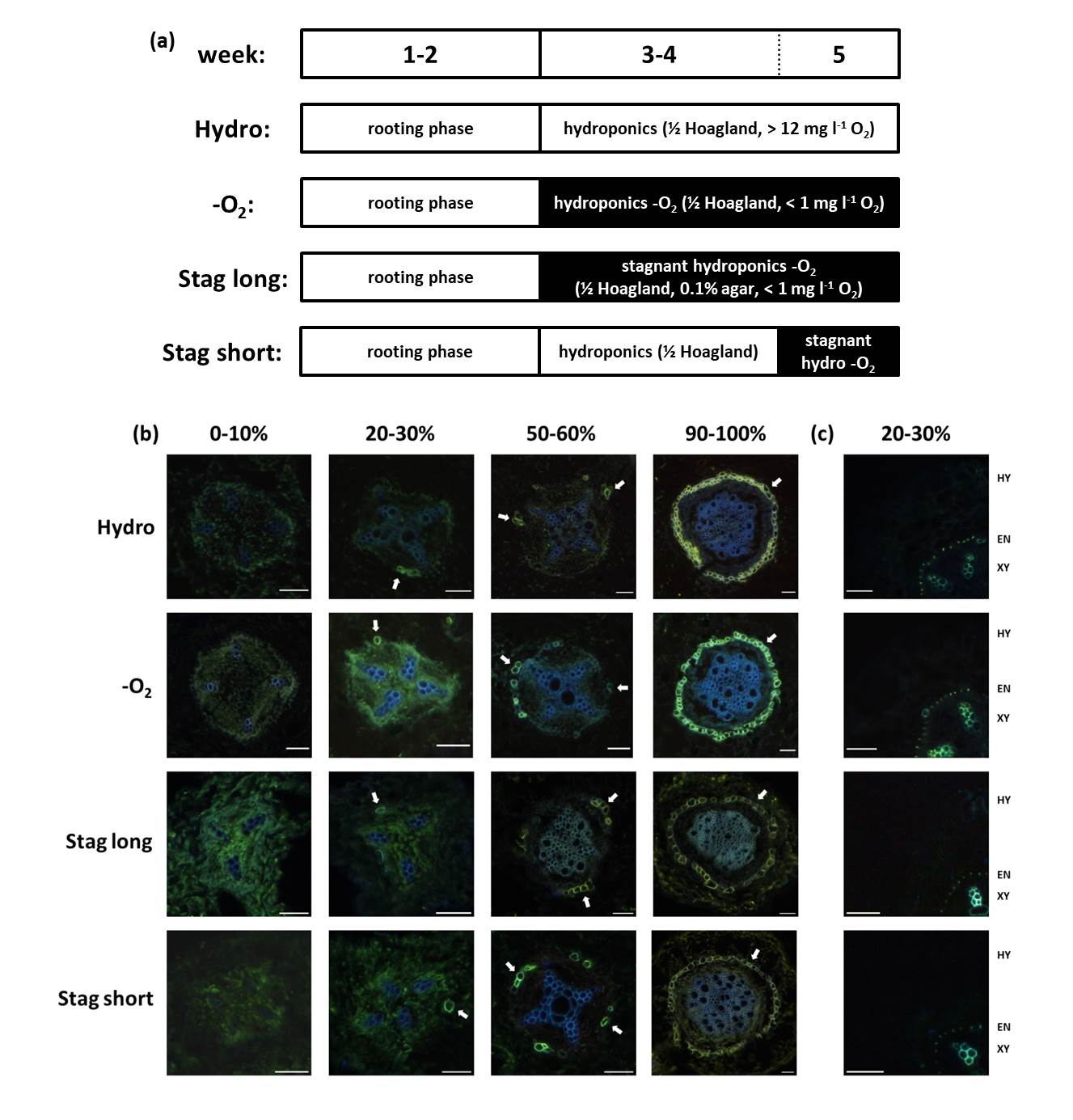


# Fig. S1 Experimental setup (a) and histochemical results (b, c) of the oxygen deficiency and stagnant conditions comparison. (a) 14- to 18-week-old plants growing in soil were dissected into stem cuttings to initiate a 2-week-long rooting phase in stagnant tap water. After stem cuttings were rooted, they were assigned to the different compared conditions (‘Hydro’ = hydroponics, ‘‑O_2_’ = oxygen deficiency, ‘Stag long’ = stagnant conditions for 3 weeks, ‘Stag short’ = stagnant conditions for 1 week). Hydroponic cultivation served as control for the comparison. Five stem cuttings were combined to yield one biological replicate and one biological replicate was examined before choosing one of the compared conditions. ½ Hoagland nutrient solution (Hoagland and Arnon 1950) was used in each experiment, either aerated in hydroponics, fumigated with gaseous nitrogen to achieve oxygen deficiency, or supplemented with 0.1% agar before fumigation with gaseous nitrogen to generate stagnant conditions. (b, c) For the suberin lamellae and Casparian band development, relative distances from the root apex (0%) to the root base (100%) are given. (b) Suberin lamellae deposition was visualized using fluorol yellow 088 applied to the whole root cross-section. However, since no suberin staining was ever achieved in the hypodermis of roots of any experiment, the figure focuses on the endodermal suberin lamellae development. Arrows indicate the onset of suberization (20-30%), transition into patchy suberization (50-60%), and almost full suberization (90-100%) of the endodermis. Scale bars = 100 µm. (c) Casparian band development was visualized using berberine-aniline blue applied to the whole root cross-section. An early exodermis formation (hypodermis with Casparian bands) was not observed in roots of this comparison (see HY indicator on the right). The endodermal Casparian bands of roots of all experiments developed highly comparable to that of the hydroponic control (see EN indicator on the right). Scale bars = 100 µm. n = 6 or more roots. EN = endodermis, EX = exodermis, HY = hypodermis, XY = xylem


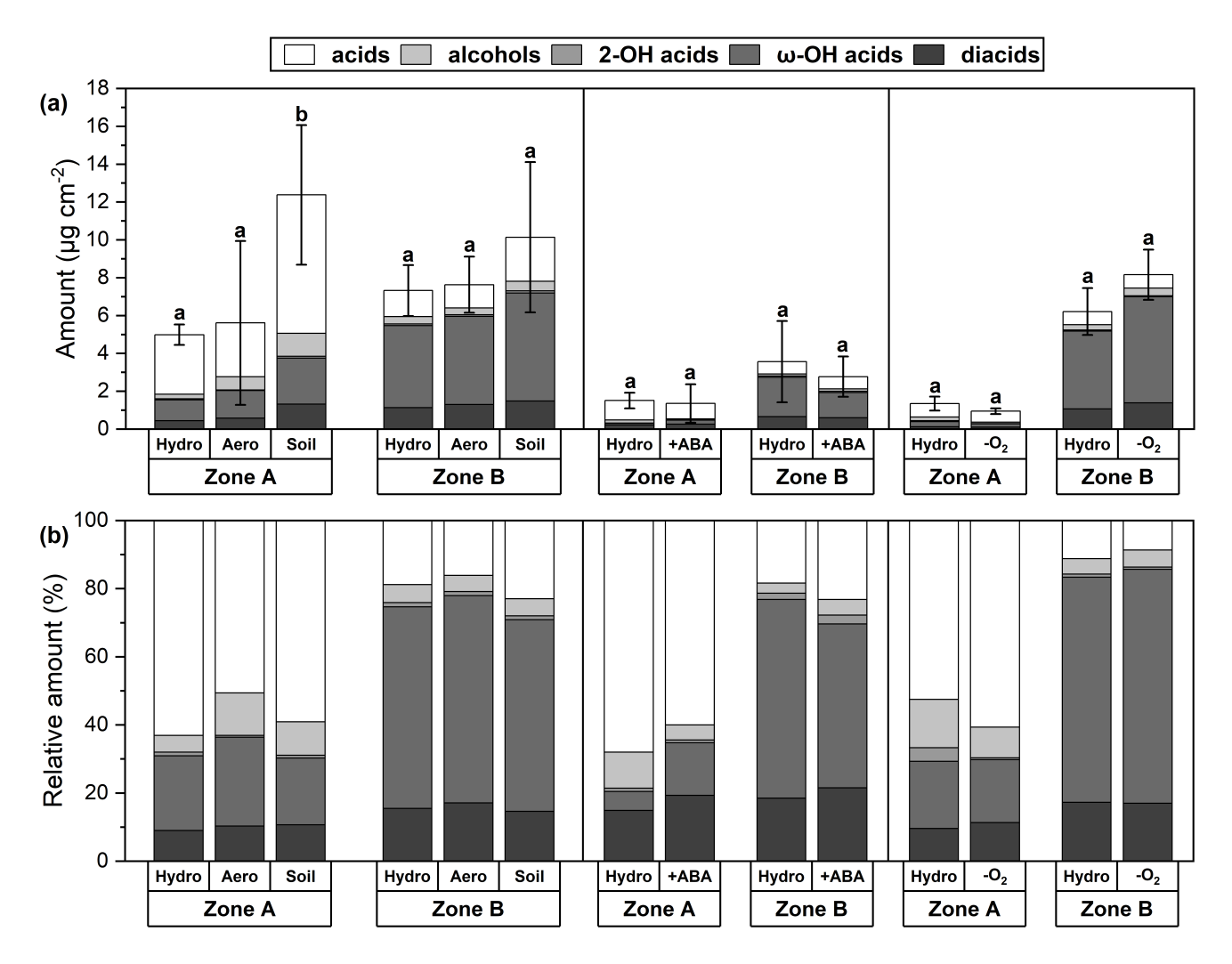


# Fig. S2 Absolute (a) and relative (b) amounts of the aliphatic suberin functional groups primary fatty acids (acids), primary fatty alcohols (alcohols), 2-hydroxy fatty acids (2-OH acids), ω-hydroxy acids (ω-OH acids), and α,ω-dicarboxylic acids (α,ω-diacids) after plant growth in different cultivation conditions and abiotic stress treatments. Experiments included hydroponic (‘Hydro’), aeroponic (‘Aero’), and soil (‘Soil’) cultivation as well as abscisic acid (‘+ABA’) and oxygen deficiency (‘‑O_2_’) stress treatment. Hydroponic cultivation served as control for all other experiments. Roots were divided into the functional Zone A (0-27.5% relative root length) and Zone B (27.5-100% relative root length). Amounts were related to the endodermal surface area. Means with standard deviations are shown. n = 4 biological replicates. Differential letters indicate significant differences at P < 0.05


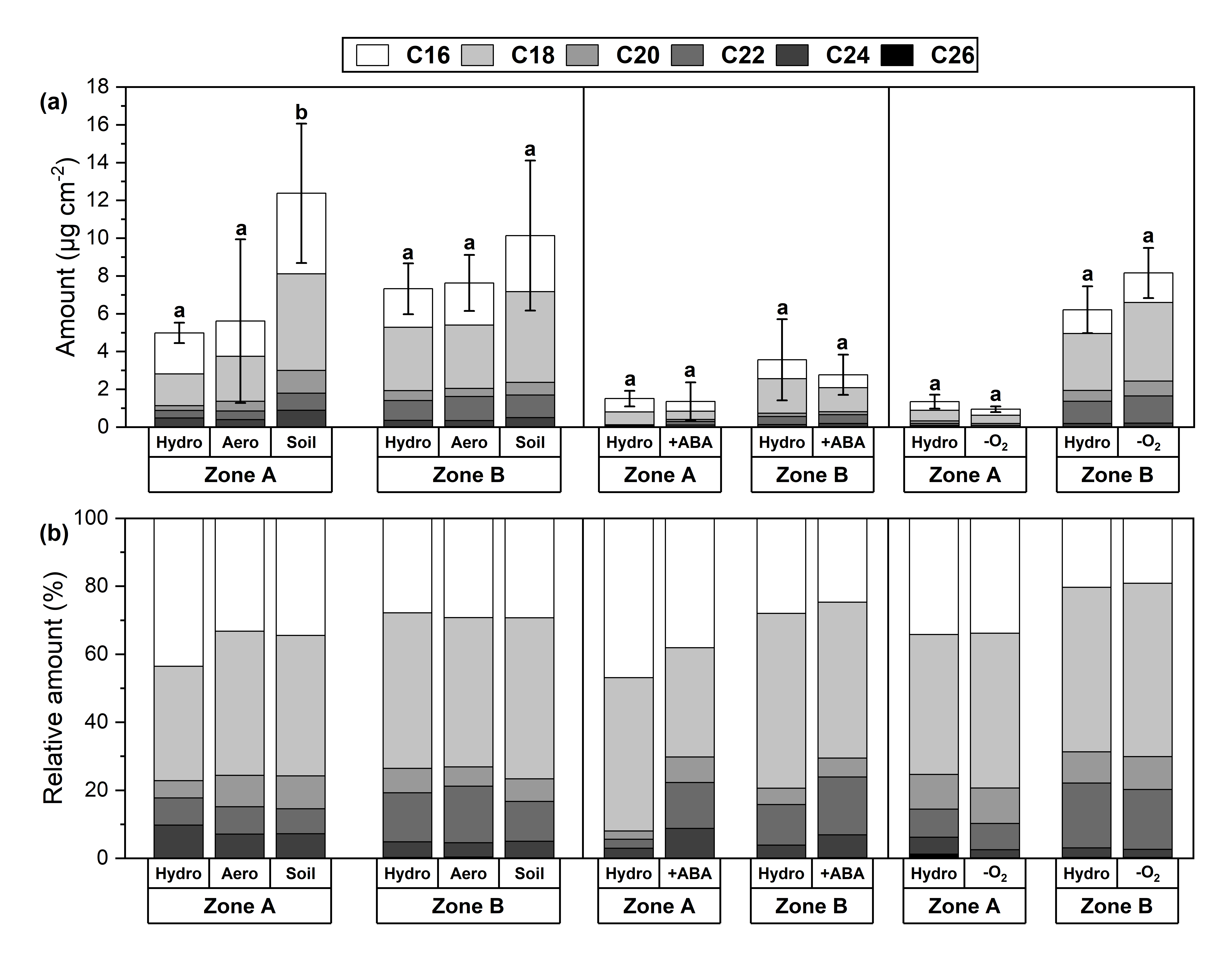


# Fig. S3 Absolute (a) and relative (b) amounts of the aliphatic suberin carbon chain length (C16-C26) distribution after plant growth in different cultivation conditions and abiotic stress treatments. Experiments included hydroponic (‘Hydro’), aeroponic (‘Aero’), and soil (‘Soil’) cultivation as well as abscisic acid (‘+ABA’) and oxygen deficiency (‘‑O_2_’) stress treatment. Hydroponic cultivation served as control for all other experiments. Roots were divided into the functional Zone A (0-27.5% relative root length) and Zone B (27.5-100% relative root length). Amounts were related to the endodermal surface area. Means with standard deviations are shown. n = 4 biological replicates. Differential letters indicate significant differences at P < 0.05.
